# Effects of Tau and Sampling Frequency on the Regularity Analysis of ECG and EEG Signals Using ApEn and SampEn Entropy Estimators

**DOI:** 10.3390/e22111298

**Published:** 2020-11-14

**Authors:** Ricardo Espinosa, Jesica Talero, Alejandro Weinstein

**Affiliations:** 1 Department of Biomedical Engineering, Universidad ECCI, Biomedical Applications EMB-IEEE, Bogotá 111311, Colombia; jesical.talerom@ecci.edu.co; 2 Center of Research and Development in Health Engineering, Valparaiso University, Valparaíso 2362905, Chile; alejandro.weinstein@uv.cl

**Keywords:** electrophysiological signals, nonlinear signals, entropy, sampling frequencies, time delay

## Abstract

Electrocardiography (ECG) and electroencephalography (EEG) signals provide clinical information relevant to determine a patient’s health status. The nonlinear analysis of ECG and EEG signals allows for discovering characteristics that could not be found with traditional methods based on amplitude and frequency. Approximate entropy (ApEn) and sampling entropy (SampEn) are nonlinear data analysis algorithms that measure the data’s regularity, and these are used to classify different electrophysiological signals as normal or pathological. Entropy calculation requires setting the parameters *r* (tolerance threshold), *m* (immersion dimension), and *τ* (time delay), with the last one being related to how the time series is downsampled. In this study, we showed the dependence of ApEn and SampEn on different values of *τ*, for ECG and EEG signals with different sampling frequencies (*F_s_*), extracted from a digital repository. We considered four values of *F_s_* (128, 256, 384, and 512 Hz for the ECG signals, and 160, 320, 480, and 640 Hz for the EEG signals) and five values of *τ* (from 1 to 5). We performed parametric and nonparametric statistical tests to confirm that the groups of normal and pathological ECG and EEG signals were significantly different (*p* < 0.05) for each *F* and *τ* value. The separation between the entropy values of regular and irregular signals was variable, demonstrating the dependence of ApEn and SampEn with *F_s_* and *τ*. For ECG signals, the separation between the conditions was more robust when using SampEn, the lowest value of *F_s_*, and *τ* larger than 1. For EEG signals, the separation between the conditions was more robust when using SampEn with large values of *F_s_* and *τ* larger than 1. Therefore, adjusting *τ* may be convenient for signals that were acquired with different *F_s_* to ensure a reliable clinical classification. Furthermore, it is useful to set *τ* to values larger than 1 to reduce the computational cost.

## 1. Introduction

The health status of a patient can be evaluated through the signals generated by the body. These signals are captured by different biomedical technologies and provide relevant information that is interpreted by a clinician. Based on these signals, the clinician can then propose a treatment to restore the patient’s health. One of the most popular techniques to capture biological signals is the placement of electrodes on the skin’s surface. These electrodes allow for the recording of the sum of action potentials generated by excitable cells (muscle and neuronal) in the body’s specific area. These signals are amplified, filtered, processed, and finally visualized and recorded in a digital medium or printed. Several physiological systems can be evaluated through these records. The most common of these electrophysiological signals are electrocardiographic (ECG) and electroencephalographic (EEG).

ECG signals provide information about the activity and function of the heart. Under normal conditions, the ECG signal presents waves with specific characteristics that are fully reported in the scientific and clinical literature. Arrhythmias are generated by a deterioration of the patient’s cardiac system or as a symptom of another underlying disease. The ECG signal can be described through its spectral characteristics, its morphology, and its regularity. Changes in these characteristics can help identify arrhythmias [1]. With the increase in deaths due to heart disease, it is paramount to develop techniques to detect heart pathologies with high precision during the clinical exploration [2].

EEG signals serve to analyze a patient neuronal activity using electrodes placed on the scalp. EEG recordings allow physicians to evaluate the patient while conscious or during sleep. The EEG allows for the evaluation of pathological states such as seizures, epilepsy, and other degenerative diseases. The EEG can be analyzed in the time and the frequency domains [3].

Traditional analysis methods provide superficial information about the biological system dynamics that generate the electrophysiological signals. Amplitude-based methods (such as mean, rectification, and root mean square) and spectrum analysis-based methods (such as Fourier transform and power spectral density) have been successfully used in the past. However, they have several disadvantages. For example, signals with very small amplitudes are more sensitive to noise contamination from surrounding radio transmissions, cables pickup, and artifacts due to patient movement [4]. Algorithms based on amplitude or frequency can be highly affected when the signal is contaminated with noise from various sources. Perhaps the main disadvantage of traditional algorithms based on amplitude or frequency is that they cannot widely describe the system’s dynamics since they are based on a linear paradigm, where small changes in the input produce small changes in the output [5]. Many characteristics that best describe the system are hidden under this approach.

Current technologies and measurement systems have allowed us to better understand the elements and their way of interaction that make up the human body’s systems. Likewise, the evolution of processing methods has made it possible to obtain more coherent information regarding the phenomena present in biological systems. However, there is still a long way to find the best tools to quantify complex phenomena effectively [6]. Physiological systems expose complex nonlinear dynamics. They consider inputs dependent on time and also inputs with random characteristics (described in this way because many details of biological interaction are still unknown) that produce irregular outputs, which have to be analyzed using nonlinear time series methods [5].

A significant number of methods have been developed to analyze nonlinear signals and characterize a biological system’s dynamic behavior. Nonlinear approaches, such as effective correlation dimension, correlation density, Hurst exponent, Lyapunov exponents, approximate entropy (ApEn), and sampling entropy (SampEn), have been applied to electrophysiological signals such as ECG and EEG [7,8,9]. This study focuses on ApEn and SampEn because numerous studies that use these estimators have emerged in recent years, both at the research-theoretical level and at the level of technological development [10].

Statistical estimators, such as entropy, are useful to characterize and analyze ECG and EEG signals. ApEn, proposed by Pincus [11,12], quantifies a time series’s regularity, particularly in nonlinear dynamic signals such as physiological signals. This method allows for distinguishing significant and subtle differences in the regularity of the data. Although ApEn is a widely used estimator, it strongly depends on the length of the recorded data. The ApEn algorithm takes segments of length *m* samples along a time series of length *N*. Each segment serves as a template for comparison with other segments of length *m* from the time series. The similarity or closeness between this template segment and the remaining segments is calculated as the conditional probability that the signal is the same under the same similarity criteria for both *m* and *m* + 1 points. This result is used as a measure of regularity [12]. In the process, a self-comparison occurs because each template vector is compared with itself to avoid evaluating the logarithmic function at zero. For this reason, ApEn generates an inconsistency in the measure of regularity that may be more noticeable when the temporal signal is short [13]. Although this bias could be eliminated if self-coincidences are excluded, this is not an alternative in ApEn.

Richman and Moorman [13] proposed the SampEn estimator as an improvement over the ApEn estimator. This improvement is based on the fact that SampEn does not generate self-comparison bias. The SampEn algorithm shows greater consistency and less dependence on the length of the data [13]. It is also more precise than ApEn, and this is essential in the study of electrophysiological time series. The calculation is expressed as ln(A/B), where A is the total number of matches in the next sample *m* + 1, and B is the total number of matches in the pattern of length *m*. The expression A/B corresponds to the conditional probability that two sequences within the same similarity criterion (tolerance *r*) for *m* points remain within *r*, at the next point (*m* + 1) [13].

In general, entropy algorithms take segments of *m* samples of the signal, called patterns, and compare them with the remaining signal (taking segments of the same length *m*). The comparison is made by measuring the Euclidean distance between the standard and the signal samples. The pattern and the segment are similar when the distances are less than a threshold *r* (or tolerance parameter). Finally, the similarity probability is calculated. Low entropy values indicate that several similarities were found, that is to say, the signal is very regular. High entropy values indicate a few similarities, that is, the signal is irregular [12,13]. Entropy algorithms are insensitive to the amplitude and frequencies of the signal. However, they show a dependence on the length of the data. When the signal length is too short, it is difficult to obtain reliable entropy values [14]. In this regard, the parameter *τ*, or time delay, corresponds to a time delay for subsampling the time series. Based on this parameter, the computation of ApEn and SampEn is performed from the first sample, and subsequently, each *τ*-th sample. 

The right choice of *τ* helps with the analysis of the data. However, to find the best choice for *τ* is not easy. Suppose *τ* is small, and the acquisition time is also minimal. In that case, neighboring data are similar, with little variation, which does not provide much information to understand the system under study. Moreover, they generate a large amount of data for each second of recording, increasing the processing time and computational cost. If *τ* is very large, the successive elements are already almost independent, the risk of loss of relevant information is high, and the processed data does not reliably represent the system’s response under study. The first zero crossing of the signal’s autocorrelation function and the mutual information are processing methods that help estimate a value of *τ* that allows for a balance between these two extremes [5,6]. Commonly, a unit delay (*τ* = 1) is used. Kaffashi et al. [15], in 2008, considered the effect of the delay time *τ* in the estimation of ApEn and SampEn. They reported that a *τ* = 1 is sufficient to generate good estimates of ApEn and SampEn for a time series whose autocorrelation function decays rapidly. However, for a time series where the autocorrelation function decays slowly, the entropy estimate can be confusing [15]. Other researchers showed that a higher *τ* value would quantify the regularity of a long-range nonlinear time series [16,17]. 

The methods applied in the nonlinear analysis of physiological signals show great potential in obtaining quantitative information for medical interpretation, which is relevant for the diagnosis of diseases. However, prior treatment of the data with traditional methods, such as Fourier analysis [18], should be guaranteed. To ensure that the digitally acquired data effectively represents the physical signal, the sampling theorem, also known as the Nyquist theorem, must be fulfilled. This theorem prescribes a condition for the sampling frequency *F_s_*.

The parameters for calculating the ApEn and SampEn entropy (tolerance threshold *r* [19], immersion dimension (*m*) [20], and time delay tau (*τ*) [15]) are described in the literature. However, little is said about the setting of these parameters for ECG and EEG signals acquired under different technologies. In particular, there are no recommendations to achieve more efficient estimations to save time, get stable entropy values, and reduce the computational cost while considering that not all technologies provide the same values of *F_s_* and gain (*G*). Entropy estimators with the same values of *r*, *m*, and *τ* (usually *τ* = 1) are often applied to signals from different sources and acquisition conditions. The following are some situations currently ignored in the literature: (i) Researchers can acquire ECG and EEG signals in the laboratory where the investigator can select *F_s_* and the gain. (ii) Often, the researcher must use fixed parameters (*F_s_* and *G*) given by the laboratory’s technology. (iii) The researcher must simply adapt to signals with different recording times, *F_s_*, and also *G*, already recorded or available in databases. It is necessary to find out whether the entropy value is the same for a signal with different acquisition characteristics (e.g., different *F_s_*) using the same parameters of *r*, *m*, and *τ*. Although the answer can be intuited, in this work, we propose a systematic study focused on the effects of *τ* in the estimation of the regularity in normal and pathological ECG and EEG signals, using ApEn and SampEn. The study shows variations in entropy estimations and provides relevant information to researchers who use ECG or EEG entropy to classify patients as healthy or sick with a different *F_s_*.

## 2. Materials and Methods 

### 2.1. Databases for ECG and EEG

**Figure 1:**
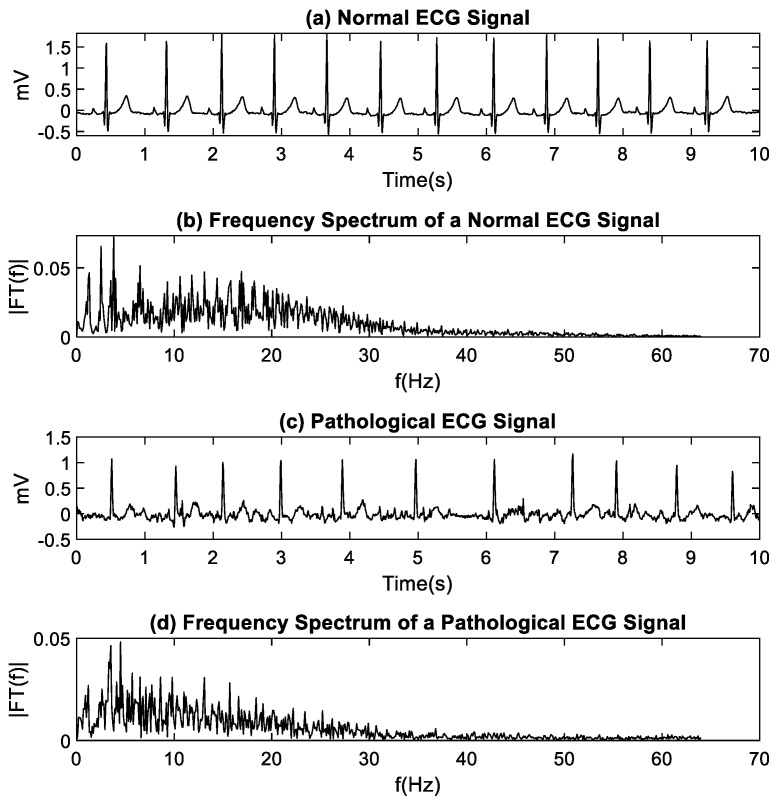
(**a**) Normal electrocardiography (ECG) signal; (**b**) Normal ECG spectrum; (**c**) Pathological ECG. (**d**) Pathological ECG spectrum.

Normal and pathological human electrophysiological signals (ECG, EEG), obtained from PhysioNet [21], were used. Normal ECG signals (subjects corresponding to healthy controls) were obtained from the PAF Prediction Challenge Database [22], which contains sets of records. Each set stores two 30-min signals, sampled at 128 Hz. From there, 20 sets were downloaded, and the first 10 s of the first signal of the set were kept. Figure 1a shows a normal ECG signal with its respective frequency spectrum in Figure 1b, showing that the relevant frequency components are below 40 Hz. Pathological ECG signals were obtained from the AF Termination Challenge Database [23], which contains sets of records. Each set stores two ECG signals with atrial fibrillation. Each signal corresponds to a 1 min segment, sampled at 128 Hz. Twenty sets were downloaded, and 10 s of the first signal in the set were kept. Figure 1c,d shows a pathological ECG signal and its respective frequency spectrum.

**Figure 2:**
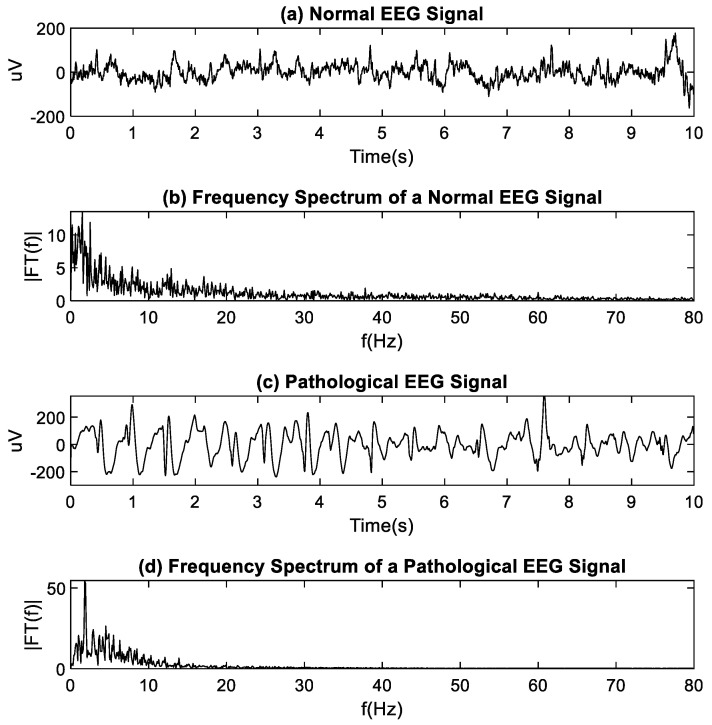
(**a**) Normal electroencephalography (EEG); **(b**) Normal EEG spectrum; (**c**) Pathological EEG; (**d**) Pathological EEG spectrum.

Normal EEG signals were obtained from the EEG Motor Movement/Imagery Dataset, which consists of more than 1500 EEG recordings between 1 and 2 min in duration. Signals were obtained from 109 volunteers, using a BCI2000 64-channel EEG system, with a sampling frequency of 160 Hz. EEG recordings correspond to several experimental conditions: 2 “baseline” or “baseline tests” of 1 min (eyes open, eyes closed) and 4 “tasks” or tests of 2 min (opening and closing the left or right fist, imagining opening and closing the left or right fist, opening and closing both fists or both feet, imagining opening and closing both fists or both feet) in several repetitions [24]. From this database, 20 signals from healthy patients (eyes open) were downloaded, and 10 s of each bipolar signal generated by the difference between the Cz-Pz electrodes were kept. Figure 2a,b shows a normal EEG signal and its frequency spectrum, respectively. 

Pathological EEG signals were obtained from the CHB-MIT Scalp EEG Database. The database contains electroencephalographic records of pediatric subjects with intractable seizures. Each case is made up of between 9 and 42 sets of records, sampled at 256 Hz. Each set contains 24–26 EEG signals [25]. Twenty recordings from different patients were downloaded, and 10 s of the bipolar Cz-Pz signal were kept. The selected electrodes (Cz-Pz) show epileptiform graphoelements present in epileptic seizures [26]. These electrodes were located on top of the brain area comprising the mid-parietal, frontal, and temporal-temporal regions. Figure 2c,d shows the pathological EEG signal and its spectrum, respectively. The characteristics of the ECG and EEG signals used in this study are summarized in Table 1.

### 2.2. Resampling of ECG and EEG Signals

The 40 ECG signals (20 normal and 20 pathological) initially had *F_s_* = 128 Hz. These signals were resampled to evaluate the effect of *F_s_* in the estimation of entropy, using linear interpolation. Sampling frequency was increased to *n* * *F_s_*, with *n* set to 1, 2, 3, and 4. The entropy values ApEn and SampEn were calculated by varying the parameter *τ* from 1 to 5. Changes of *F_s_* were made for each value of *τ*. A total of 1600 datasets were processed, i.e., 2 (ApEn and SampEn) × 40 (normal ECG signals and pathological ECG signals) × 4 (different *F_s_*) × 5 (different *τ* values).

The 40 EEG signals (20 normal and 20 pathological) had different sampling frequencies. For this reason, before calculating ApEn and SampEn, pathological EEG signals with *F_s_* = 256 Hz (original) were downsampled to *F_s_* = 160 Hz to match the normal EEG recordings, which were sampled at 160 Hz [24]. Once the normal and pathological EEG signals had the same sampling frequency (*F_s_* = 160 Hz), the value of ApEn and SampEn was calculated, with the parameter *τ* varied from 1 to 5. For each change in *F_s_*, 5 changes were made for *τ* for each EEG signal, without breaking Nyquist’s theorem. A total of 1600 data were processed, i.e., 2 (ApEn and SampEn) × 40 (normal EEG signals and pathological EEG) × 4 (different *F_s_*) × 5 (different *τ* values).

### 2.3. Approximate Entropy (ApEn) and Sampled Entropy (SampEn)

ApEn and SampEn estimate the regularity of a time series. Their calculation depends on the conditional probability that two similar sequences, with a tolerance of *r* and of length *m*, remain similar in the next *m* + 1 sample. ApEn and SampEn are smaller when there are many similar sequences and a few nonsimilar sequences. Low ApEn and SampEn values reflect high regularity. In this work, the following parameters were used to calculate the entropy: (immersion dimension) *m* = 2 and (tolerance threshold) *r* = 0.2 of the signal’s standard deviation. These values coincide with those suggested by Pincus et al. [12] in their work on ApEn and Richman and Moorman [13] in their study on SampEn, among many other studies that used the same values. The ApEn and SampEn algorithms are shown below.

#### 2.3.1. Algorithm ApEn

Step 1: For a time series of *N* samples {u(n) : 1 ≤ n ≤ N}, fix a value of *m* and *τ* integers and *r*. 

Step 2: Form the sequence of the vector $X^{m}\left(1\right)$ to $X^{m}\left(N-\left(m-1\right)\tau \right)$ as

(1) $$X^{m}\left(i\right)=\left\{u\left(n\right),\;u\left(n\;+\;1\tau \right),\;\ldots ,\;u\left(n\;+\left(m\;-\;1\right)\tau \right)\right\},\;n=\;1,\;\ldots ,N\;-\left(m-\;1\right)\tau .$$

Step 3: Define the distance between $X^{m}\left(i\right)$ and $X^{m}\left(j\right)$ as:

(2) $$d_{ij}=d\left[X^{m}\left(i\right),X^{m}\left(j\right)\right]=\max\left\{\left|u\left(i+k\tau \right)-\;u\left(j+k\tau \right)\right|:0\leq k\;\leq m-1\right\}$$

Step 4: Estimate the probability $C_{i}^{m}\left(r\right)$ as:

(3) $$C_{i}^{m}\left(r\right)=\left(N-(m-1\right)\tau {)}^{-1}\sum_{j=1}^{N-\left(m-1\right)\tau }\theta \left(d_{ij}-r\right),$$

where *θ* is the Heaviside function defined as: $\theta =\{\begin{array}{c} \;1,\;si\;d_{ij}\leq r\;\\ 0,\;si\;d_{ij}>r \end{array}$

Step 5: Calculate

(4) $$\Phi^{m}\left(r\right)={\left(N-\left(m-1\right)\tau \right)}^{-1}\sum_{i=1}^{N-\left(m-1\right)\tau }lnC_{i}^{m}\left(r\right)$$

Step 6: Increase *m* in 1, and find $C_{i}^{m+1}\left(r\right)$ and $\Phi^{m+1}\left(r\right)$

Step 7: For a finite sequence of length N, define

(5) $$ApEn\left(m,r,N\right)=\Phi^{m}\left(r\right)\;-{\;\Phi }^{m+1}\left(r\right).$$

#### 2.3.2. Algorithm SampEn

Step 1: For a time series of *N* samples {u(n) : 1 ≤ n ≤ N}, fix a value of *m* and *τ* integers and *r.*

Step 2: Form the sequence $X^{m}\left(i\right)=\{u\left(n\right),\;u\left(n+\tau \right),\;\ldots ,u(n+\left(m-1\right)\tau )\}$ for *n* = *i* = 1,…, *N*-(*m*-1)*τ*).

Step 3: Define the distance between $X^{m}\left(i\right)$ and $X^{m}\left(j\right)$ as

(6) $$d_{ij}=d\left[X^{m}\left(i\right),X^{m}\left(j\right)\right]=\max\left\{\left|u\left(i+k\right)-\;u\left(j+k\right)\right|:0\leq k\;\leq \left(m-1\right)\tau \right\}$$

Step 4: Define $B_{i}^{m}$ as ${\left(N-\left(m+1\right)\tau \right)}^{-1}$ times the number of vectors $X^{m}\left(j\right)$ within *r*, which remain close to $X^{m}\left(i\right)$, where *j* takes the rank of 1 to *N-mτ*, and *j ≠ i* to exclude the auto matches, then it is defined as

(7) $$B^{m}=\;{\left(N-m\tau \right)}^{-1}\;\sum_{i=1}^{N-m\tau }B_{i}^{m},\;$$

where $B_{i}^{m}=\;{\left(N-\left(m+1\right)\tau \right)}^{-1}\;\overset{N-m\tau }{\underset{j=1,j\neq i}{\sum }}\theta \left(r-d\left[X^{m}\left(i\right),X^{m}\left(j\right)\right]\right)$

Step 5: Change the embedding dimension from *m* to *m* + 1, repeat from Step 2 to Step 4, then get $A^{m}$:

(8) $$A^{m}=\;{\left(N-m\tau \right)}^{-1}\;\sum_{i=1}^{N-m\tau }A_{i}^{m},$$

where $A_{i}^{m}=\;{\left(N-\left(m+1\right)\tau \right)}^{-1}\;\overset{N-m\tau }{\underset{j=1,j\neq i}{\sum }}\theta \left(r-d\left[X^{m+1}\left(i\right),X^{m+1}\left(j\right)\right]\right)$

Step 6: Define

(9) $$A^{m}=\;{\left(N-m\tau \right)}^{-1}\;\sum_{i=1}^{N-m\tau }A_{i}^{m},$$

with $B^{m}$ and $A^{m}$ being the probability that 2 sequences coincide for *m* points and *m* + 1 points, respectively.

### 2.4. Statistical Analysis of Entropy in ECG and EEG Signals

Results obtained with ApEn and SampEn were compared between normal and pathological signals (ECG, EEG) using parametric and nonparametric statistical tests. The t-student test was applied to compare the groups that presented normal distribution and equality of variances, and the Mann–Whitney U test for the groups that did not comply with the normality and variance test.

## 3. Results

**Figure 3:**
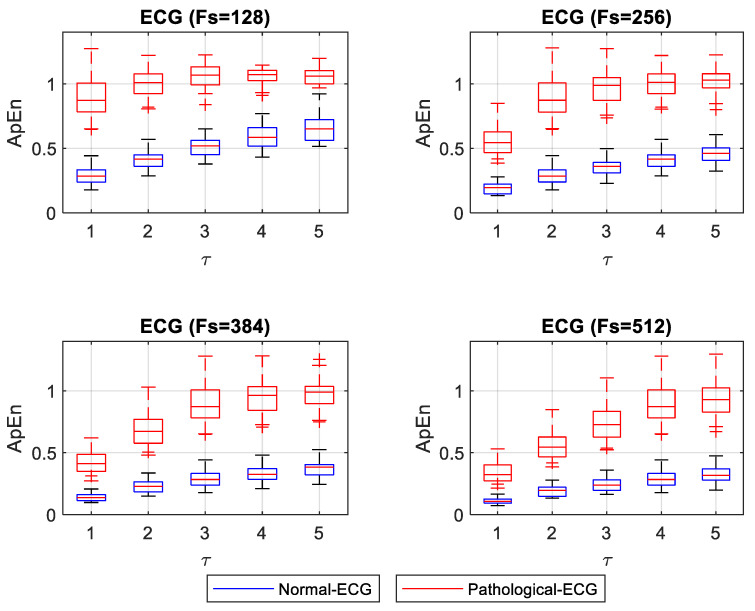
Box plot of ApEn for different *F_s_* and *τ* of normal and pathological ECG signals.

In this section, we present graphs of the ApEn and SampEn entropy estimation for normal and pathological ECG signals and normal and pathological EEG signals for different values of *F_s_* and *τ*. *F_s_* values start from a minimum value, complying with the Nyquist Theorem, and then increases in *n* * *F_s_*, with *n* = 1, 2, 3, and 4. Values of *τ* increased by one unit, from 1 to 5. Entropies of 40 ECG signals were estimated (20 normal and 20 pathological). Entropy values for normal ECG signals were concentrated in the low entropy values, confirming the ECG signal’s regularity. Entropy values for pathological ECG signals were concentrated at high entropy values. The nonparametric Mann–Whitney U test was applied to compare normal and pathological ECG for each *F_s_* and *τ*. For the ApEn case, normality evaluation, using the Shapiro–Wilk test, showed that the group of ApEn values for normal-ECG signals did not have a normal distribution. In contrast, the group of ApEn values for pathological-ECG signals follows a normal distribution. Figure 3 shows the box plot for ApEn values for different *F_s_* and *τ*. The statistical test showed significant differences (*p* < 0.05) between normal and pathological ECG for each *F_s_* and *τ*.

**Figure 4:**
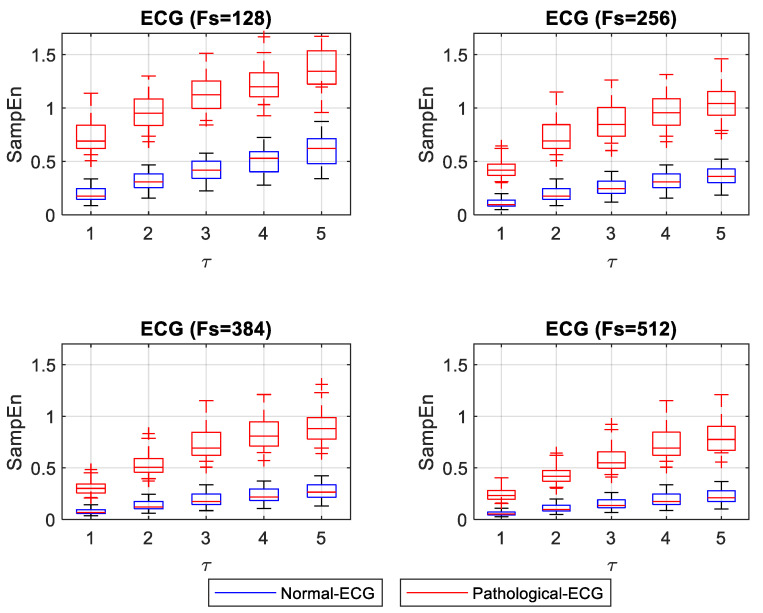
Box plot of SampEn for different *F_s_* and. *τ* of normal and pathological ECG signals.

SampEn did not show a normal distribution for the normal ECG signals. In contrast, it did show a normal distribution for the pathological signals. Figure 4 shows the box plot of SampEn for different *F_s_* and *τ*. The statistical test showed significant differences (*p* < 0.05) between normal and pathological ECG for each *F_s_* and *τ*.

**Figure 5:**
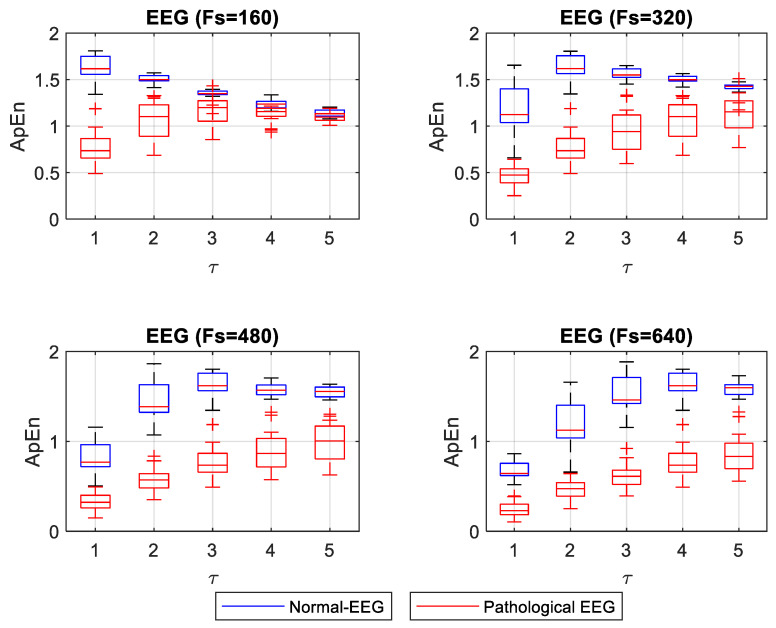
Box plot of ApEn for different *F_s_* and *τ* of normal and pathological EEG signals.

ApEn and SampEn entropy values were estimated for 40 EEG signals (20 normal and 20 pathological). Entropy values for normal EEG signals concentrated on high entropy values, showing that these signals are irregular. Entropy values for pathological EEG signals concentrated at low entropy values. A nonparametric Mann–Whitney U statistical test was applied to compare normal and pathological EEG for each *F_s_* and *τ*. For the ApEn case, normality evaluation, using the Shapiro–Wilk test, showed that 40% of the groups of ApEn values for normal-EEG signals followed a normal distribution. In comparison, 95% of the ApEn group of values for pathological-ECG signals showed a normal distribution. In the case where both the normal and pathological EEG ApEn value groups had a normal distribution, the Levene test was applied to evaluate the equality of the variances. The test showed differences in variances, confirming the result of the Mann–Whitney U test. Figure 5 shows the box plot for ApEn values for different *F_s_* and *τ*. The statistical test showed significant differences (*p* < 0.05) between normal and pathological EEG for each *F_s_* and *τ*. However, for *F_s_* = 160 and *τ* = 5, it had a *p* = 0.045, showing closeness between normal and pathological EEG.

**Figure 6:**
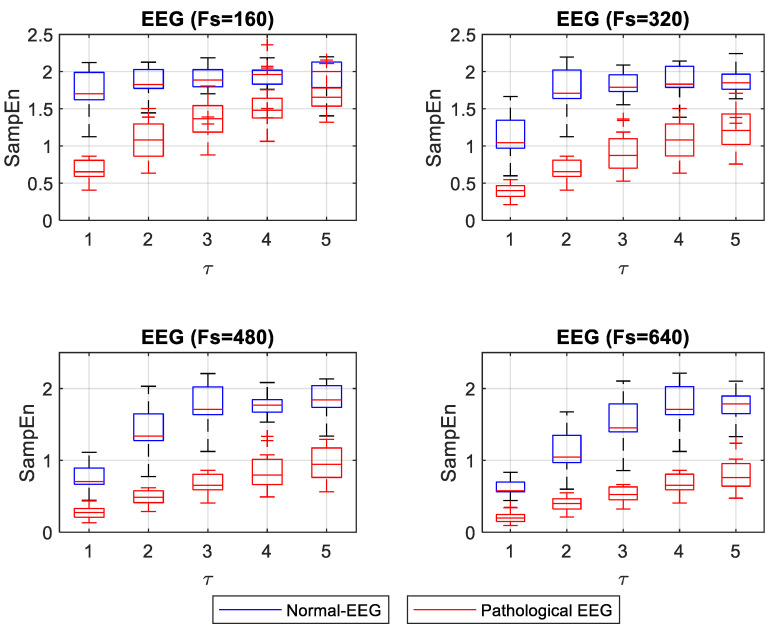
Box plot of SampEn for different *F_s_* and *τ* of normal and pathological EEG signals.

For the case of SampEn for EEG signals, the Shapiro–Wilk test showed that 90% of the groups of SampEn values for normal-EEG signals followed a normal distribution, while all groups of SampEn values for pathological-EEG signals followed a normal distribution. For cases in which both the normal and pathological EEG SampEn values groups had a normal distribution, the Levene test was applied to evaluate the variances’ equality. The test showed that 50% of the groups fulfilled the equality of variances. For these groups, the t-student test was applied. The remaining groups were tested with the Mann–Whitney U test. Figure 6 shows the box plot for SampEn values at different *F_s_* and *τ* values. The statistical test showed significant differences (*p* < 0.05) between normal and pathological EEG in each *F_s_* and *τ*.

## 4. Discussion

ECG and EEG signal analysis is prevalent in assessing the health condition of a patient. This prevalence explains why many researchers have focused on applying processing methods to extract characteristics that reveal information from the body’s system under study. Processing methods for nonlinear signals, such as entropy, are useful to classify ECG and EEG signals as normal or pathological, based on their regularity. The algorithms to calculate ApEn and SampEn are widely used today, and the values for parameters *r*, *m*, and *τ* are widely discussed in the scientific literature. Different reports explain the appropriate values of *r*, *m*, and *τ*, assuming that an ECG or EEG signal’s natural characteristics are maintained during the acquisition process. However, this is not entirely true because several factors may change during the signal acquisition, such as the gain, the sampling frequency, the noise level, among other factors. Sampling frequency *F_s_* is a critical factor in obtaining appropriate signals representing the system’s response under evaluation. A decreased *F_s_* that is below the Nyquist Theorem’s conditions can distort the signal and give false information from the source system. A high *F_s_* may end in high-frequency noise induced by radio transmission interference, in addition to resulting in a large amount of data that could overwhelm the processing stage. Therefore, the values of the parameters *r*, *m*, and *τ* should be adjusted depending on the signal acquisition conditions. Subtle changes in the resulting entropy values between signals acquired from different sources could be of diagnostic value in the classification of these signals as normal or pathological.

A numerical experiment was designed to study changes in ApEn and SampEn values for ECG and EEG signals with different *F_s_*, representing different acquisition systems. The experiment consisted of calculating ApEn and SampEn for normal and pathological ECG and EEG signals. Figure 3 and Figure 4 show that normal ECG signals have low entropy values. This result means that they exhibit some regularity. In contrast, entropy values for pathological ECG signals have higher values. This result confirms the observations made in 2018 by Horie et al., where they examined SampEn values in ECG signals from patients with atrial fibrillation (AF) and patients without arrhythmia. In this work, they found that the SampEn values were higher for patients with AF than for patients without arrhythmia. Horie used a data acquisition frequency of *F_s_* = 200 Hz, and the results of entropy SampEn were compared with *τ* = 1 and *τ* = 5. In both cases, SampEn was different between patients with AF and without arrhythmia [27]. Based on these findings, it could be interpreted that the entropy values remained stable with changes in *τ*. However, Figure 3 and Figure 4 show that entropy values, both for normal and pathological ECG signals for *F_s_* set to 256, slightly increased as *τ* increased. Although normal and pathological conditions remain significantly different (*p* < 0.05), with *τ* = 1 the entropy values between normal and pathological were closer than for other values of *τ*. An adjustment of *τ* in the calculation of entropy and *F_s_* in data acquisition could be appropriate to guarantee a more stable distinction between signals from patients with AF and without arrhythmia.

The most substantial distinction between normal and pathological ECG signals was for the smallest *F_s_* and with *τ* = 1. With increasing values of *τ*, ApEn values for normal ECG signals tend to rise, indicating that the signals are less regular. The opposite happens with higher *F_s_*, where a *τ* = 1 shows that pathological ECG signals become more regular, approaching the normal ECG values. Values of *τ* greater than 1 with higher *F_s_* showed separation between the normal and pathological ECG. Moreover, they shower more stable ApEn values. SampEn results showed a similar separation for all *τ* values evaluated with the minimum *F_s_*. SampEn values increased slightly with increasing values of *τ*. However, for higher *F_s_*, the SampEn values of normal and pathological signals were too close for low *τ* values. As *τ* increased, the distinction between normal and pathological ECG signals improved. 

For the EEG signals, ApEn and SampEn entropy values were high for the normal condition and low for the pathological condition (see Figure 5 and Figure 6). Having regular and irregular signals allows for observing the effect of different *F_s_* and *τ* values on the entropy capacity to distinguish the normal from pathological cases. These changes are not discussed in the literature thoroughly. Mesin in 2018 proposed to modify ApEn for estimating the regularity of EEG signals to correct the dependence on the length of the data; this work evidenced the convenience of increasing the *F_s_* to obtain more reliable entropy values [17]. In this present study, we confirmed that increasing *F_s_* improves entropy measurements when one wants to differentiate the normal from the pathological EEG signals. When ApEn is estimated with a lower *F_s_*, it is convenient to use *τ* = 1. The increases in *τ* make the concentration of ApEn values (high and low) closer, thus making it very difficult to distinguish between normal and pathological signals (*p* = 0.045 for a *τ* = 5 and an *F_s_* = 160). However, for higher *F_s_* it may be more advisable to use a *τ* between 1 and 3, where a better separation between the high and low values of ApEn is shown. In Figure 6, SampEn shows a more stable separation. The SampEn value of the pathological EEG signals increases slightly with increasing *τ*. With a lower *F_s_*, normal-EEG values remain almost stable, and a *τ* = 1 is advisable to make a significant separation between normal and pathological EEG. With increasing *F_s_*, it is convenient to use a *τ* greater than 1 if a more stable separation between regular and irregular signals is desired. 

In summary, our results highlight the importance of appropriately selecting *F_s_* and *τ* to be able to differentiate robustly between the healthy and the pathological cases using the entropy of ECG and EEG signals. This entropy estimation allowed us to characterize signals with AF and epileptic patterns. These results suggest that this approach can also be applied to other cardiac and neurological pathologies. 

## 5. Conclusions

The use of ApEn and SampEn in the analysis of nonlinear ECG and EEG signals have had an important reception in current research. According to their regularity, ECG or EEG signals can be classified as normal or pathological. This behavior makes entropy a quantity of interest for clinical uses. To compute ApEn and SampEn values, parameters *r* (tolerance threshold), *m* (immersion dimension), and *τ* (time delay) are set beforehand. The first two (*r* and *m*) are widely discussed in the literature, and their effects on the entropy value are known. However, *τ* is also a factor that significantly affects the value of entropy. Many studies set *τ* = 1, and in many others, its value is not discussed.

Parameters *F_s_* and *τ* are familiar. They are related to the electrophysiological signal’s resampling, but they are taken into account at different times. *F_s_* is considered during the acquisition of the signal. It is frequently not a value that the researcher can manipulate since it is generally fixed in the acquisition device. Furthermore, the value of *F_s_* may be different from one device to the next. On the other hand, *τ* is set during the ApEn or SampEn entropy calculation, and the researcher can purposely manipulate this parameter. 

When the researcher sets the parameters *r*, *m*, and *τ*, it is common to standardize these parameters for signals from different sources (a laboratory or from a digital repository), assuming that the entropy value will not depend on the signal origin. For ECG and EEG signals, a stable entropy calculation must be ensured to provide reliable values. This is particularly important for clinical interpretation, where a patient is being classified as healthy or sick.

In this study, we show the effect of *τ* and *F_s_* on the computation of ApEn and SampEn. We show the convenience of adjusting values of *τ* depending on the nature of the signal (ECG or EEG) and the acquisition characteristics, particularly the sampling frequency, to obtain more reliable regularity measures able to distinguish between normal and pathological signals.

This study’s most important observations are as follows. SampEn and ApEn can be used to significantly differentiate between regular signals (normal-ECG and pathological-EEG) and irregular ones (pathological-ECG and normal-EEG) by varying *τ* and *F_s_*. However, for ApEn, with a minimum *F_s_*, a value of *τ* = 1 is advisable when it is desirable to distinguish between normal and pathological ECG. In contrast, with a higher *F_s_*, the use of *τ* greater than 1 is advisable because it maintains a distinction between normal and pathological ECG while reducing the data processing burden. SampEn for the analysis of ECG signals showed a slight increase in entropy values with the increasing values of *F_s_* and *τ*. The distance between the SampEn values of normal and pathological ECG signals was similar to the minimum *F_s_* for all values of *τ*. The optimal parameters to achieve a large separation, observed between normal and pathological ECG entropy values, were obtained using SampEn with a low *F_s_* (128 or 256 Hz) and a large value of *τ* (*τ* = 5 or *τ* = 4).

In the analysis of ApEn and SampEn measurements for normal and pathological EEG, a value of *τ* = 1 is convenient when using a minimum *F_s_*. An increase of *τ* in this condition (low *F_s_*) degrades the entropy value, bringing the normal and pathological EEG measurements closer together. This makes the distinction difficult. With increasing values of *F_s_*, it is advisable to use a value of *τ* greater than 1 to obtain a clear separation of normal and pathological EEG. However, a high value of *τ* (4 or 5) could corrupt the values, especially for the regular signals (pathological-EEG). The optimal parameters to achieve a large separation, observed between normal and pathological EEG entropy values, were obtained using SampEn with a high value of *F_s_* (480 or 640 Hz) and a large value of *τ* (3, 4, or 5).

It is of great value to recognize the appropriate adjustments of parameter *τ* in calculating ECG and EEG signals’ entropy to have consistent regularity measures while reducing processing cost, especially for long-duration recordings. 

In this study, we analyzed the separation between entropy values of normal and pathological signals (AF for the ECG case and epileptic seizures for the EEG case). However, for other cardiac or neurological pathologies, the separation between normal and pathological may depend on the patient’s conditions. This separation is a characteristic to be explored, which may improve the clinical identification of groups of patients with different conditions.

## Figures and Tables

**Table 1:** Characteristics of ECG and EEG signals used in this study.

Signals	No.	Databases	Time	*F_s_*
Normal ECG	20	The PAF Prediction Challenge Database	10 s	128 Hz
Pathological ECG	20	The AF Termination Challenge Database	10 s	128 Hz
Normal EEG	20	EEG Motor Movement/Imagery Dataset	10 s	160 Hz
Pathological EEG	20	CHB-MIT Scalp EEG Database	10 s	256 Hz
